# The Optimization of a Label-Free Electrochemical DNA Biosensor for Detection of *Sus scrofa* mtDNA as Food Adulterations

**DOI:** 10.3390/bios13060657

**Published:** 2023-06-15

**Authors:** Yeni Wahyuni Hartati, Irkham Irkham, Iis Sumiati, Santhy Wyantuti, Shabarni Gaffar, Salma Nur Zakiyyah, Muhammad Ihda H. L. Zein, Mehmet Ozsoz

**Affiliations:** 1 Department of Chemistry, Faculty of Mathematics and Natural Sciences, Universitas Padjadjaran, Bandung 45363, Indonesia; irkham@unpad.ac.id (I.I.); iissumiati638@gmail.com (I.S.); santhy.wyantuti@unpad.ac.id (S.W.); shabarni.gaffar@unpad.ac.id (S.G.); salma17005@mail.unpad.ac.id (S.N.Z.); muhammad21444@mail.unpad.ac.id (M.I.H.L.Z.); 2 Department of Biomedical Engineering, Near East University, Mersin 10, 99138 Nicosia, Turkey; mehmet.ozsoz@neu.edu.tr

**Keywords:** electrochemical DNA biosensor, streptavidin–biotin, label-free, pork, *Sus scrofa*

## Abstract

Fast, sensitive, and easy-to-use methods for detecting DNA related to food adulteration, health, religious, and commercial purposes are evolving. In this research, a label-free electrochemical DNA biosensor method was developed for the detection of pork in processed meat samples. Gold electrodeposited screen-printed carbon electrodes (SPCEs) were used and characterized using SEM and cyclic voltammetry. A biotinylated probe DNA sequence of the Cyt b *S. scrofa* gene mtDNA used as a sensing element containing guanine substituted by inosine bases. The detection of probe-target DNA hybridization on the streptavidin-modified gold SPCE surface was carried out by the peak guanine oxidation of the target using differential pulse voltammetry (DPV). The optimum experimental conditions of data processing using the Box–Behnken design were obtained after 90 min of streptavidin incubation time, at the DNA probe concentration of 1.0 µg/mL, and after 5 min of probe-target DNA hybridization. The detection limit was 0.135 µg/mL, with a linearity range of 0.5–1.5 µg/mL. The resulting current response indicated that this detection method was selective against 5% pork DNA in a mixture of meat samples. This electrochemical biosensor method can be developed into a portable point-of-care detection method for the presence of pork or food adulterations.

## 1. Introduction

The technology of species identification in food products for determining food adulteration led to the development of many advanced analytical methods and offers fast and authentic results, one of which is the DNA-based method [1,2,3]. Furthermore, DNA is a stable molecule that supports the analysis of processed materials by heating or the use of raw materials. DNA is present in most cells and can be easily extracted from any type of tissue. Some of the additional advantages of a DNA-based analysis are simplicity, speed, and specificity. The PCR method is the most commonly used method for this purpose and for detecting the presence of pork in food, as reported in the last three years [4,5,6,7,8,9,10,11,12,13,14].

The Cyt b gene of pork is often used to identify pork DNA in food products by using PCR techniques [12]. The selection of this Cyt b coding gene sequence is unique in that it has a conserve part and, thus, can be used to determine the kinship of an organism [15]. This gene also provides a large number of copies for each cell, which can increase sensitivity in a significant real-time assay and contribute to survival when the tissue is subjected to extreme conditions such as sterilization or heating [15,16].

DNA-based biosensors offered a standard test for the presence of pork; this test is more time- and cost-efficient than PCR. Several DNA biosensor methods were reported for the detection of pork content in samples, including optical DNA biosensors using gold nanoprobes to assay the pork contaminants in food or in the raw and processed meat [17,18,19,20,21,22], the luminescence optical biosensor [23], fiber optic genosensors [24], and fluorescence [25,26] that can detect a small quantity of pork.

The electrochemical biosensor was widely developed for DNA detection due to its numerous advantages such as high level of sensitivity, good specificity, fast response time, and ease of miniaturization [27]. An electrochemical DNA biosensor using gold-modified SPCE electrodes with an AuNP/DNA bioconjugate was developed to detect mtDNA *S. scrofa* with methylene blue as an indicator, and can detect 10% content in a mixture of processed meat [28]. The use of a ruthenium hexamine molecule as an indicator was also reported [29], along with that of a ruthenium (II) complex, [Ru(bpy)_2_PIP]^2+^, (bpy = 2, 2′-bipyridine, PIP = 2-phenylimidazo [4,5-f][1,10-phenanthroline]) as the label redox complex [30]. An electrochemiluminescence method was also developed for the detection of pork with a low limit of detection [31].

On the other hand, a label-free DNA-based electrochemical biosensor was reported for distinguishing pork, beef, and chicken as raw meat, by using graphene-modified SPCEs [32]. The label-free electrochemical DNA technique is based on the intrinsic electroactive properties of the DNA molecule. The nitrogen base that is most easily oxidized is guanine. This detection technique reduces the need for additional indicators and detection steps. To eliminate the guanine oxidation signal on the probe, the guanine on the probe is replaced with an inosine base that is similar in structure to the guanine base but is not easily oxidized and will form hydrogen bonds with cytosine with its complement pair; therefore, the oxidation signal only arises from guanine in the target DNA sequence that complements the DNA probe [33].

In this study, the label-free detection of *S. scrofa* Cyt B DNA was carried out using gold-electrodeposited screen-printed carbon electrodes. The streptavidin–biotinylated DNA probe system was used for the immobilization process, and signal transduction detection was determined from the targeted guanine signals. The sequence of probes used in this study consisted of a 27-nts probe designed with guanine-substituted inosine. Optimization of experimental conditions was carried out using the Box–Behnken design method.

## 2. Materials and Methods

### 2.1. Materials

Chloroauric (HAuCl_4_), potassium ferricyanide (K_3_[Fe(CN)_6_].3H_2_O), sodium hydroxide (NaOH), and potassium chloride (KCl) were obtained from Merck (Darmstadt, Germany). Buffered phosphate saline (PBS) (VWR Amresco Life Science, Wayne, NJ, USA), pork probe DNA 5′- 5Bio-ITA TTI ATA CCA ATC ACT AIC ATC ATC-3’ (I is Inosine), and complementary pork target DNA 5’-GAT GAT GCT AGT GAT TGG TAT CAA TAC -3’, beef target DNA; 5′-CTA CTA CGA TCA CTA ACC ATA GTT ATG-3′, chicken target DNA; 5′-ACA AGG CTA ACA CCC CTT CTC CTA ATC CTA-3′. (Integrated DNA Technologies, Singapore); restriction enzyme BamHI (New England Biolabs, Ipswich, MA, USA); agarose; SyBR^®^ Safe DNA Gel Stain (Thermo Scientific, Waltham, MA, USA); Blue/Orange 6× loading dye (Promega, Madison, WI, USA); SiZerTM-1000 DNA Marker (Intron Biotechnology, Gyeonggi, Republic of Korea); double distilled water (PT Ikapharmindo Putramas, Jakarta, Indonesia); streptavidin (Promega, Madison, WI, USA); and QI-Aprep^®^ Spin Miniprep Kit (Cat. 27104) (Qiagen, Hilden, Germany) were used. Next, for the DNA extraction, the meat samples of pork, beef, and chicken were obtained from a local supermarket.

The morphology of the electrode surfaces was characterized using scanning electron microscopy (SEM) (Inspect S50, FEI, Hilsboro, OR, USA), a screen-printed carbon electrode (SPCE) 4 mm (GSI Technologies, Sunnyvale, CA, USA), and a Zimmer Peacock potentiostat were used for the electrochemical measurement.

### 2.2. Electrodeposition of Gold onto SPCE

The SPCE surface was dripped with 40 µL of HAuCl_4_·3H_2_O (100 µg/mL), and electrodeposition was carried out with differential pulse voltammetry (DPV) at the potential range of −0.5 V to +0.5 V and a scan rate of 8 mV/s [34]. After electrodeposition, the electrodes were rinsed with double distillation water and dried in air and were then characterized using SEM and electrochemically using impedance spectroscopy (EIS) and DPV based on the 10 mM K_3_[Fe(CN)_6_] redox system in 0.1 M KCl with a potential range of −0.5 to 0.5 V at a scan rate of 8 mV/s.

### 2.3. Modification of Gold-SPCE with Streptavidin (SA)-Biotynlated DNA Probe

The gold–SPCE was rinsed with double distilled water, and then, 20 µL of SA (100 μg/mL) was dropped onto the surface, and the modified SPCE was incubated at 4 °C for 30, 60, or 90 min. Thereafter, they were rinsed thrice with PBS (pH 7.4). Next, 40 µL of biotinylated probe DNA (1.0 µg/mL) was added to the surface of the electrodes, and the electrodes were incubated for 10, 15, or 30 min at 25 °C. After the incubation, the electrodes were washed again three times with PBS (pH 7.4) and stored at room temperature.

### 2.4. Optimization of Box–Behnken Experimental Design

Factors such as SA incubation time (X1), biotinylated probe DNA concentration (X2), and target hybridization time (X3) were selected as factors to be optimized in the experiment. Each factor was designed to have three different levels, namely the lowest (−1), medium (0), and the highest (+1), as shown in Table 1.

The experiment was carried out 15 times (Table S1), and the measurement results were then processed. The optimum value of each factor was determined using the experimental design of Minitab 19. General experiments were carried out by adding 20 µL of SA on the gold–SPCE surface, incubating the electrode for 90 min, adding 20 µL of probe DNA (0.5 µg/mL) and 20 µL of target DNA (1 µg/mL), and then incubating the electrode again for 10 min.

### 2.5. Determination of DNA Biosensor Response to Synthetic Target DNA

Gold–SPCE/SA/biotinylated probe DNA was dripped with 40 µL of target DNA with a variation of DNA concentration from 0.5, 0.75, 1.0, 1.25, to 1.5 µg/mL and incubated at the optimal hybridization time. The guanine oxidation signal was measured using a differential pulse voltammetry analysis technique in the potential range of −0.2 V to +1.4 V at the scan rate of 8 mV/s. Furthermore, the electrodes were immersed in PBS (pH 7.4) for 10 s and stored at room temperature. 

### 2.6. Extraction of DNA

The DNA of pork, chicken, and beef was isolated using QIAprep^®^ Spin Miniprep Kit (Thermo Scientific, Waltham, MA, USA). In total, 25 mg of the sample was mashed into a 2 mL microtube. Then, 250 µL of buffer P1 (resuspension buffer) was added and inverted. Next, we added 250 µL of buffer P2 (lysis buffer) and inverted the microtube again until the solution became clear (the lysis process was confirmed to last not more than 5 min). Then, 350 µL of the inversion buffer N3 (neutralization buffers) was added 4–6 times. Thereafter, centrifugation was performed for 10 min at 13,000 rpm.

Next, as much as 800 µL of the supernatant fluid was transferred into the QIAprep 2.0 spin column and was then centrifuged for 1 min, and the liquid in the collection tube was discarded. Then, we added 500 µL of the buffer PB (phosphate buffer), centrifuged the sample for 1 min, and discarded the liquid in the collection tube. The baby spin column was added with 750 µL of the buffer PE (wash buffer) containing ethanol and was again centrifuged for 1 min. The liquid in the collection tube was discarded, and the sample was centrifuged for 1 min. Next, we placed the spin column in a new 1.5 mL microtube and added 50 µL of the buffer EB (elution buffer is 10 mM TrisCl, pH 8.5). The sample was then incubated for 1 min and centrifuged for 1 min. The DNA isolates were then stored at −20 °C.

### 2.7. Characterization of DNA Extracts with BamHI Restriction Enzymes

In total, 1 µg of the DNA isolates from the total DNA extracts produced were reacted with 2 µL of the buffer RE 10× (Restriction Enzyme, pH 8.0). Then, 0.2 µL of acetylated BSA and 1 µL (10 units/µL) of the BamHI enzyme were added. Next, we added with nuclease-free water until the total volume reached 20 µL. The isolates were then incubated at 37 °C for 4 h. The cutting results were then characterized using agarose electrophoresis, which is explained in more detail in Supplementary Materials (Figures S1 and S2, Table S2).

### 2.8. Determination of DNA Biosensor Current Response to DNA Samples

**Figure 1:**
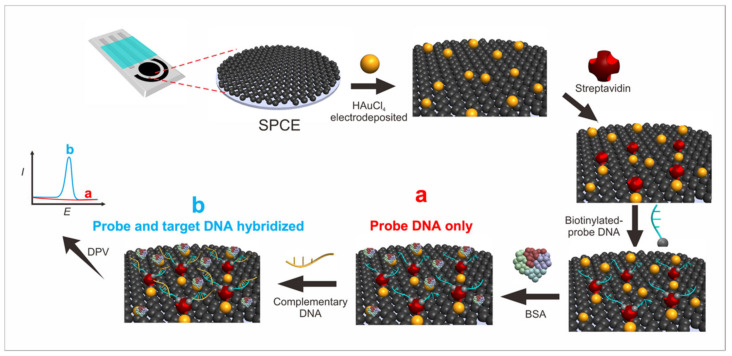
Schematic representation of the biosensor used in this study. SPCE was first modified by gold nanoparticles (AuNPs) using HAuCl_4_ by electrodeposition. Then, SPCE is immobilized with streptavidin, which will bind specifically and with high affinity to the biotinylated DNA probe. BSA was then added to the surface as a blocking agent that can prevent non-specific binding with the surface of electrode. The DNA probe then will hybridize with the DNA target (complementary DNA). Then, characterization of the biosensor using the DPV and EIS methods will show peaks of guanine oxidation currents that will be observed after adding the target.

The restricted DNA sample (target) was diluted ten times to reach a total volume of 200 µL and denatured at 95 °C for 1 min in a water bath. Next, the DNA isolates were transferred to an ice bath for 10 s. Then, approximately 40 µL of the DNA isolates was stapled on the gold–SPCE/SA/probe DNA and incubated for 5 min. Next, the electrode was immersed in a PBS (pH 7.4) solution for 10 s, and a guanine oxidation signal was observed on the differential pulse voltammetry in the potential range of −0.2 V to +1.4 V at the scan rate of 8 mV/s. The biosensor scheme used in this study is shown in Figure 1, and the steps of each procedure are detailed below.

### 2.9. Aplication Biosensor for Real Sample

Meat samples were extracted directly from meatballs, both individual and mixed samples. DNA isolates from restriction using BamHI were diluted 10 times. Then, 40 µL of the sample was on the SPCE/Au/SA/DNA probe-biotin surface. Sample DNA isolates containing pig DNA will specifically hybridize with probe DNA. The response of the DNA biosensor from isolate DNA samples was characterized by DPV.

## 3. Results and Discussion

### 3.1. Immobilization of Biotinylated Probe DNA and Hybridization of Probe-Targeted DNA

SPCE was modified by gold nanoparticles by means of HAuCl_4_ electrodeposition, and streptavidin was then immobilized on the SPCE–Au surface through physical adsorption by dropping it on the electrode surface. It is possible to conjugate a molecule with a large molecular weight (for example, protein) to gold particles through the adsorption method [35].

The biotinylated DNA probe was immobilized at the SPCE–Au/SA electrode by using the streptavidin–biotin system. This streptavidin–biotin system directed the DNA probe to a good orientation when immobilized at the electrode. The biotin molecule interacted with various residues in the β-barrel of streptavidin to form a series of hydrogen bonds. This interaction then caused the sequencing of the surface loops that covered the entire biotin molecule, thereby increasing the strength of the bond. Hydrogen bonding in streptavidin–biotin occurred between the serine amino acid residues on streptavidin with the amine group in the bicyclic ring in biotin and between the aspargin amino acid residues on streptavidin with the carboxylic groups on the biotin valeric acid chain.

**Figure 2:**
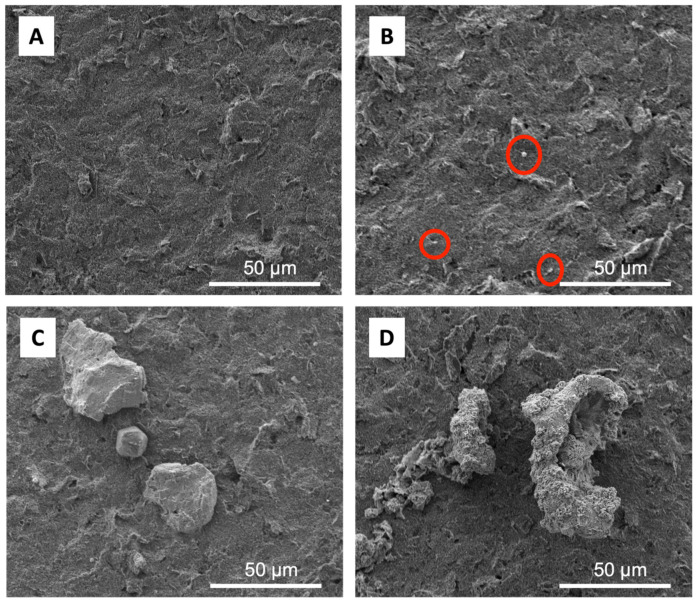
(**A**) SEM images of SPCE bare; (**B**) SPCE-AuNPs, the red circle shows the deposited AuNP; (**C**) SPCE-Au/Streptavidin; and (**D**) SPCE-Au/Streptavidin/Probe DNA.

Figure 2A shows the SEM images of SPCE before and Figure 2B shows after gold modification, where a small gold particle was observed, which is highlighted by a red circle. When the electrode was modified to gold and the addition of SA was carried out, it resulted in an electrode surface with SA particles that stuck evenly so that the distribution of the surface area on the Au–SA particles was homogeneous (Figure 2C), while further adding the probe gave a bigger and rough particle on the electrode surface (Figure 2D).

**Figure 3:**
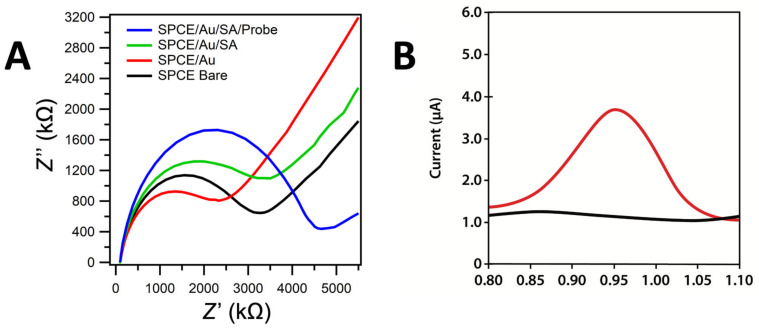
(**A**) Electrochemical Impedance Spectroscopy measurement results of 10 mM K_3_[Fe(CN)_6_] in 0.1 M KCl on the Bare SPCE (black), SPCE Au (red), SPCE/Au/Streptavidin (green), and SPCE/Au/Streptavidin/probe DNA (blue). (**B**) Differences in the peak guanine oxidation currents for inosine containing probe DNA (black line) and probe-target DNA (red line) at SPCE/Au/SA. The concentration of the DNA probe used was 1.0 µg/mL and that of the synthetic target DNA was 1.0 µg/mL while the buffer was 0.01 M PBS in pH 7.4, potential range of +0.7 to +1.2 V at the scan rate of 8 mV/s.

The characterization of immobilization with the streptavidin–biotin system was carried out using electron impedance spectroscopy (EIS) to see the dynamic of electrode resistance in each step of the modification (Figure 3A) using a Randles circuit (Figure S3) and DPV measurement of ferricynide oxidation (Figure S4). It can be seen that the resistance of electrode was decreased after the electrodeposition of gold due to the gold metallic feature, which had a high conductivity (Figure 3A, red line). Meanwhile, the resistance increased after each biomolecules modification, which indicated that a successful anchoring of the probe DNA on the Streptavidin in the surface of SPCE.

The target guanine oxidation signal on SPCE/Au/SA for biotinylated probe DNA and the hybridization of probe DNA with targeted DNA was measured by the substitution of the probe guanine with inosine. The probe DNA did not exhibit a peak of guanine oxidation because the guanine base in the probe was replaced with an inosine base, which would not exhibit an oxidation peak in the guanine oxidation region. Inosine is a nitrogenous base that has properties similar to those of guanine, which can form hydrogen bonds with cytosine with its complement pair but will not provide an oxidation signal in the guanine oxidation signal area [36]. Before adding the target, BSA was added as a blocking agent to cover the side of the modification that could lead to a non-specific binding with the target biomolecule. Figure 3B shows that the peak of the guanine oxidation current was in the range of 0.9–1.0 V [37]. The peak of the guanine oxidation current upon the addition of the probe DNA and synthetic targeted DNA was about 3.98 µA, with the concentration of the DNA probe used was 1.0 µg/mL and that of the synthetic target DNA 1.0 µg/mL. Meanwhile, probe DNA did not show any peak current of guanine due to the substitution of guanine with inosine base.

### 3.2. Optimal Experimental Conditions with Box–Behnken Experimental Design

Three factor experiments with three levels of each factor considered led to 15 runs of the experiment. These three factors were SA incubation time, probe concentration, and target hybridization time. The experiment was carried out in triplicate. The optimum value of each of these factors was determined using the Box–Behnken experimental design with the Minitab 19 program. From the processing of the response data from the measurement results of the experiment (data in the Supplementary Materials), the coefficient of the response function was obtained to predict the maximum current value. From this experiment, the following regression equation was obtained:

Y = 14.29 − 0.365 X1 + 11.48 X2 − 0.685 X3 + 0.003398 X1 × X1 − 7.08 X2 × X2 + 0.0503 X3 × X3 + 0.0268 X1 × X2 − 0.00694 X1 × X3 − 0.002 X2 × X3

where Y = current responses (µA), X1 = time of immobilization of SA (min), X2 = probe DNA concentration (µg/mL), and X3 = time of hybridization (min).

From the regression equation, we inferred that a factor with a negative coefficient caused a decrease in the response, while a factor with a positive coefficient increased the response. Factors that influenced the reduction in the response were the time of SA immobilization and the time of target hybridization. Meanwhile, the factor that affected the response increase was the concentration of the probe. The optimum conditions obtained from the results of the data processing were the SA incubation time of 90 min, the concentration of DNA probe of 1.0 µg/mL, and the target hybridization time of 5 min.

### 3.3. Calibration Curves and Analytical Parameters

The guanine and adenine bases are the most electroactive components in the DNA sequence, and guanine is more electroactive than adenine. The number of guanine and adenine bases in order the oligonucleotide reflects the peak current of the oligonucleotide. Increasing the number of guanine increasing the guanine oxidation signal [38].

**Figure 4:**
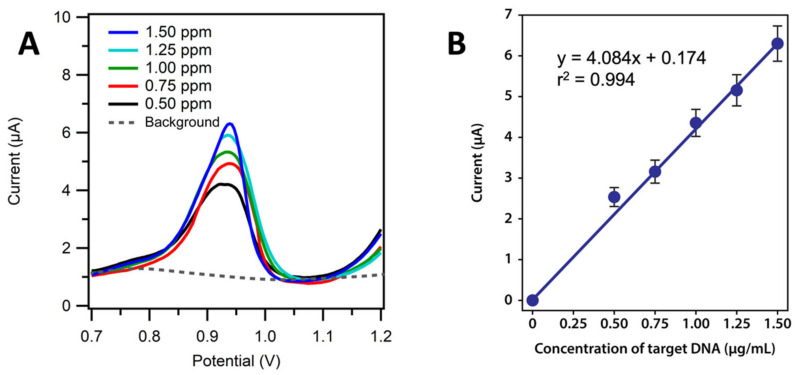
(**A**) Guanine oxidation signals of various concentration of synthetic target DNA Voltammogram of guanine oxidation differential pulses of 0.5 to 1.5 µg/mL. (**B**) Calibration curve of DNA biosensor to the concentration of synthetic target DNA for 0 to1.5 µg/mL of synthetic target DNA. The buffer was 0.01 M PBS in pH 7.4, potential range of +0.7 to +1.2 V at the scan rate of 8 mV/s.

The triplicate measurements of the various concentration of the synthetic target DNA were carried out at the optimum conditions of the experiment (0.5, 0.75, 1.0, 1.25, 1.5 µg/mL of the synthetic target DNA). Figure 4A shows the voltammogram of the various in the concentration of synthetic target DNA. The higher the target DNA concentration, the higher the peak current of target guanine that underwent an oxidation reaction to 8-oxoguanine. The target DNA concentration above 1.5 µg/mL indicates the data were outside the linear range. Figure 4B shows a linear calibration curve of 0.5–1.5 µg/mL of synthetic target DNA. The calibration curve obtained showed a linear relationship between the synthetic target DNA concentration and the peak current with the regression equation:

y = 4.0843x + 0.1744, (r^2^) = 0.994

The sensitivity of the DNA biosensor voltammetry method was expressed by the slope value of the linear regression equation, which was 4.084. The detection and the quantification limits were 0.135 µg/mL and 0.450 µg/mL, respectively. The determination of the precision, accuracy, and the recovery was performed seven times for each measurement repetition. We obtained a precision value of 96.36%, an accuracy of 99.94%, and a recovery of 91.57%.

### 3.4. Sample Analysis

**Figure 5:**
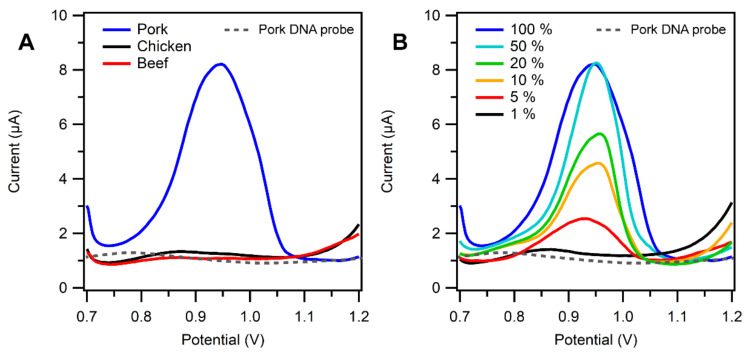
(**A**) Voltammograms of DNA samples extracted from pork, beef, and chicken, dash line pork DNA probe, blue line pork DNA Probe +pork DNA isolated target, black line pork DNA probe + chicken DNA isolated target, red line Pork DNA probe + beef DNA isolated target, (**B**) Mixed sample containing 1% to 100% of pork extracts. The buffer was 0.01 M PBS in pH 7.4, potential range of +0.7 to +1.2 V at the scan rate of 8 mV/s.

The DNA biosensor was tested against the DNA isolated from the processed meat samples, both individual and mixed samples. The DNA isolates were cut with the BamHI restriction enzyme and diluted 10 times. The use of BamHI was intended because the DNA probe sequence used in this study was the DNA sequence in the 1091 to 1110 region in the CytB gene, and the restricted side of BamHI made it easier for the pork DNA extract to produce pieces of the mtDNA region such that it closely matched the DNA probe. The DNA extract samples containing pork DNA hybridized specifically with the DNA probe (which contains Inosine instead of Guanine). The responses of the DNA biosensor to the isolated DNA sample are shown in Figure 5. The mixed meat samples contained pork, chicken, and beef; varying percentages of pork were then cooked, DNA was extracted, and the extracts were cut using the restriction enzyme BamH1. The amount of enzyme-restricted DNA extract measured was about 5 µg/mL based on the procedures listed in the determination of sample section.

**Figure 6:**
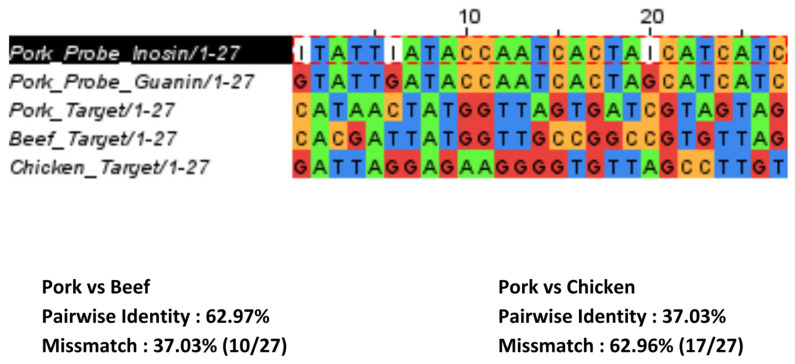
Pairing of the 27-sequence probe DNA pork used in this experiment with pork target DNA, beef target DNA, and chicken target DNA.

Figure 5 shows DPV results in the pork, beef, chicken, and those mixtures using SPCE/Au/SA/Probe for pork electrodes. Based on Figure 5A, the chicken and beef samples gave almost no response, while the pork sample gave an obvious current signal. This happened because those samples did not hybridize with the DNA probe at the electrode. This could be attributed to the fact that the DNA sequences in the cattle beef (37.03% mismatch) and the chicken (44.44% mismatch) did not match with the DNA probe sequences for pork (Figure 6). It is also important to note that even though the DNA from the meat sample was longer than those from the synthetic sample, the external guanine from the rest of the chain that did not hybridize to the probe DNA might not affect the signal of the measurement by much, due to big steric hinderance in the surface of the electrode [37].

Furthermore, a variation of pork content in the mixed pork, chicken, and beef samples (Table S6) was also investigated using DPV (Figure 5B, Table S7). The results showed that as the amount of the pork DNA increased in the mix, the oxidation current response increased. The assay revealed that the electrochemical biosensor for the detection of pork mtDNA that was developed was a selective and sensitive method because it could detect the pork DNA contaminants in the samples up to a level of 1%. The 5% pork content in the mixed sample could be distinguished from the peak current of the chicken and the beef samples, while the 1% content was still indistinguishable visually, and the peak current close to the response of chicken and beef.

The comparison of the determination of pork content in the reported DNA-based biosensor samples are shown in Table 2. Based on Table 2, this label-based biosensor work showed a limit of detection lower than the other methods for the synthetic DNA target. Although this method cannot detect to a mixture with 1% pork which was previously investigated by using an optical chemiluminescence genosensor (24), this method can still be an alternative to distinguish in processed meat sample with 5% pork content.

## 4. Conclusions

The proposed label-free electrochemical biosensor was very simple because it only detected the presence or absence of a guanine peak current after the application of the sample to the biosensor. With the streptavidin–biotin system, the DNA probe can lead well and can be used for the detection of DNA extracts after preparation with the BamHI restriction enzyme. This DNA-free label biosensor can detect pork contaminants above 1% in a processed meat mixture. Furthermore, this method can be used and developed as a method that is easy to use and miniaturize.

## Figures and Tables

**Table 1:** Three levels of factors considered in the experiment.

Factor	Unit		Level	
		−1	0	+1
Time of SA incubation	min	30	60	90
Concentration of probe DNA	µg/mL	0.5	1.0	1.5
Time of target hybridization	min	5	10	15

**Table 2:** The comparison of DNA-based biosensor in the determining of pork.

Methods	Limit of Detection		Linear Range (Μg/Ml)	Reference
	DNA (μg/mL)	In Mixture (%)		
GNP sensor (colorimetric)	6.00	20	0.3–9.0	[19]
GNP sensor (colorimetric)	4.00	10	0.4–6.0	[20]
Chemiluminescent optical fiber genosensor	2.00	1	1.0–7.7	[24]
Gold-DNA bioconjugate electrochemical biosensor	0.58	10	0.1–5.0	[28]
Graphene-based electrochemical biosensor	1.76	-	1.0–10.0	[32]
SPCE-cerium based electrochemical biosensor	1.44	-	5.0–30.0	[39]
Fluorescence-based CRISPR	2.7	-	0–200	[25]
SPCE-Graphene Acid based electrochemical biosensor	-	9	–	[40]
SPCE-gold-based electrochemical biosensor	0.135	5	0.5–1.5	This work

## Data Availability

Experimental data associated with this research are available from the authors.

## Supplementary Materials

The following supporting information can be downloaded at: https://www.mdpi.com/article/10.3390/bios13060657/s1, Figure S1: Electropherogram of sample DNA isolates on a 1% agarose gel, Figure S2: Electropherogram of isolates resulting from restriction with BamH1 enzymes, Figure S3: Schematic of Randles circuit used for the EIS measurement, Figure S4: Differential pulse voltammogram of each before and after step of electrode modification, Table S1: Box-Behnken experimental design and current result for each experiment, Table S2: Quantification results of DNA isolates using multimode, Table S3: Data on the linear regression of synthetic target DNA with DPV, Table S4: Slope value adjustment data, Table S5: Data precision, accuracy, and recovery, Table S6: Percentage Composition of Meat (Meatballs), Table S7: Peak current value from DPV measurement of mixed meat samples.
