# Supplementary material for: The Optimization of a Label-Free Electrochemical DNA Biosensor for Detection of Sus scrofa mtDNA as Food Adulterations
Source: Biosensors (Basel). 2023 Jun 15;13(6):657. doi: 10.3390/bios13060657 (PMC10296283; doi:10.3390/bios13060657)
Supplement: Supplementary file 1 [file biosensors-13-00657-s001.zip › biosensors-2400144-supplementary.pdf]

Supplementary Material

The Optimization of a Label-Free Electrochemical DNA Biosensor for Detection of *Sus scrofa* mtDNA as Food Adulterations

Yeni Wahyuni Hartati [1]*, Irkham [1], Iis Sumiati [1], Santhy Wyantuti [1], Shabarni Gaffar [1], Salma Nur Zakiyyah [1], Muhammad Ihda H.L. Zein [1], and Mehmet Ozsoz [2]

[1] *Department of Chemistry, Faculty of Mathematics and Natural Sciences, Universitas Padjadjaran, Bandung, Indonesia·*

[2] *Department of Biomedical Engineering, Near East University, Mersin 10, 99138 Nicosia, Turkey*

* Corresponding author. E-mail addresses: yeni.w.hartati@unpad.ac.id; Tel.: +628122132349

Table of contents:

(Figure S1) Electropherogram of sample DNA isolates on a 1% agarose gel.

(Figure S2) Electropherogram of isolates resulting from restriction with BamH1 enzymes.

(Figure S3) Schematic of Randles circuit used for the EIS measurement.

(Figure S4) Differential pulse voltammogram of each before and after step of electrode modification.

(Table S1) Box–Behnken experimental design and current result for each experiment.

(Table S2) Quantification results of DNA isolates using multimode.

(Table S3) Data on the linear regression of synthetic target DNA with DPV.

(Table S4) Slope value adjustment data.

(Table S5) Data precision, accuracy, and recovery.

(Table S6) Percentage Composition of Meat (Meatballs).

(Table S6) Peak current value from DPV measurement of mixed meat samples.

Box–Behnken Experimental Result

Table S1. Box–Behnken experimental design and current result for each experiment

No	X_1	X_2	X_3	Current (µA)
1	30	0,5	10	5,669
2	30	0,5	10	7,893
3	30	1,0	5	7,045
4	30	1,0	15	6,419
5	30	1,5	10	6,046
6	60	0,5	5	2,164
7	60	0,5	15	5,558
8	60	1,0	5	5,253
9	60	1,0	10	5,749
10	60	1,5	5	3,804
11	60	1,5	15	3,768
12	90	0,5	10	7,872
13	90	1,0	5	11,777
14	90	1,5	10	5,946
15	90	1,0	15	6,099

Sample Exctraction Analysis

DNA isolation went through three stages: cell lysis, precipitation, and purification. The isolated DNA was visualized and analyzed using 1% agarose electrophoresis to determine the presence of DNA, the integrity of the isolated DNA, and the purity of the DNA from RNA contaminants. The characterization with the addition of loading dye for a marker of the rate of DNA migration. Figure S4 shows the results of DNA isolation. The results of the electropherogram showed that the resulting DNA bands had smeared, indicating the presence of several isolated genomic DNA fragments.

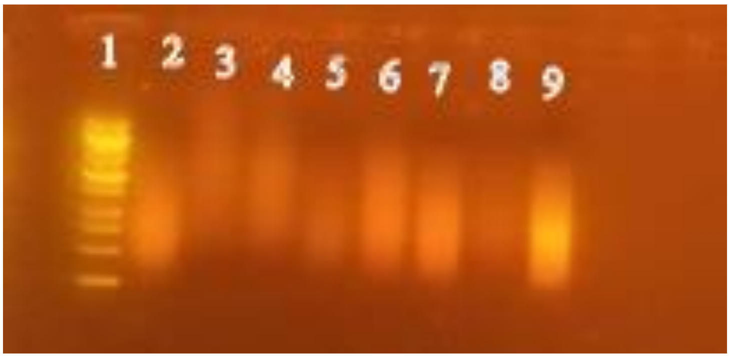

Figure S1. Electropherogram of sample DNA isolates on a 1% agarose gel. Well, 1. DNA marker 1 kb; 2. 100% chicken DNA; 3. 100% pork DNA; 4. 100% beef DNA; 5. Mixed DNA 50%; 6. Mixed DNA 20%. 7. Mixed DNA 10%; 8. Mixed DNA 5%; 9. Mixed DNA 1%

The next step was to determine the concentration and purity of the isolated DNA using a multimode spectrophotometer that had a working principle such as a UV spectrophotometer. Tests were carried out at the wavelength regions of 260 nm and 280 nm as indicators of the level of DNA purity (1.8–2.0) (Mulyani et al., 2011). The concentration of extracted DNA isolates gave a high ratio value, so that the extracted DNA isolates could be said to be pure and have no RNA or protein contamination.

The isolated DNA was then restricted using the restriction enzyme BamHI and characterized using 1% agarose electrophoresis. The restriction result is shown in Figure S5, showing the resulting smear and thin bands. Restricted DNA isolates were used to test the response and selectivity of the electrochemical DNA biosensor method.

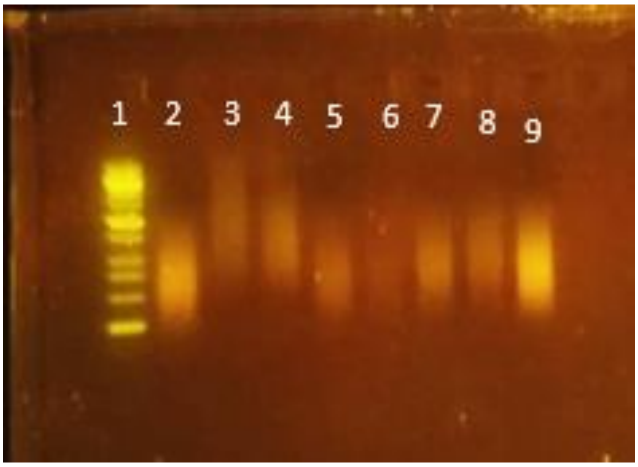

Figure S2. Electropherogram of isolates resulting from restriction with BamH1 enzymes. Well, 1. DNA marker 1 kb; 2. 100% chicken DNA; 3. 100% pork DNA; 4. 100% beef DNA; 5. Mixed DNA 50%; 6. Mixed DNA 20%. 7. Mixed DNA 10%; 8. Mixed DNA 5%; 9. Mixed DNA 1%.

Table S2. Quantification results of DNA isolates using multimode.

Isolate DNA (%)	Absorbance		Concentration (ng/μL)	Ratio
	A_{260}	A_{280}		
A 100	0.1204	0.0601	120.4	2
S 100	0.0653	0.0333	65.3	1.96
B 100	0.0396	0.0198	39.6	2
A: B: S 25: 50: 25	0.0358	0.0177	35.8	2.02
A: B: S 55: 20: 25	0.0944	0.0475	94.4	1.99
A: B: S 65: 10: 25	0.0257	0.0128	25.7	2.01
A: B: S 70: 5: 25	0.1145	0.0579	114.5	1.98

A: B: S<br>74: 1: 25	0.1119	0.0558	111.9	2.01

A = chicken, B = pork, S = beef

Modified Electrodes Characterization

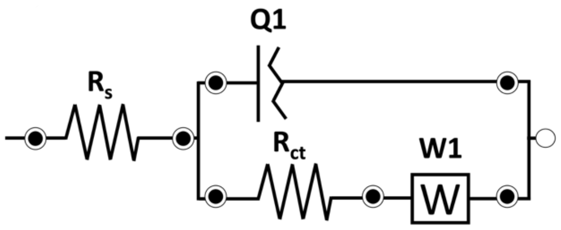

Figure S3. Schematic of Randles circuit used for the EIS measurement.

No	Consentration target (x) μg/mL	I (y) /μA	İ (y) /μA	Standard deviation	$(xi)^2$	(x-ẋ)	(y- ȳ)	$(x- ẋ)^2$	$(y- ȳ)^2$	(x-ẋ) y-ȳ)
1	0	0	0	0	0	-0.833	-3.578	0.694	12.804	2.982
		0								
		0								
2	0.5	2.982	2.520	0.438	0.25	-0.333	-1.058	0.111	1.120	0.353
		2.112								
		2.466								
3	0.75	2.955	3.162	0.651	0.563	-0.083	-0.418	0.007	0.175	0.035
		2.640								
		3.892								
4	1.0	4.214	4.341	0.282	1	0.167	0.762	0.028	0.580	0.127
		4.145								
		4.664								
5	1.25	2.929	5.175	1.25	1.563	0.417	1.602	0.175	2.565	0.667
		4.546								
		6.615								
6	1.5	7.733	6.270	1.803	2.25	0.667	2.692	0.444	7.245	1.794
		6.831								
		4.247								
Total	5		21.47		5.625			1.458	24.490	5.958
Average	0.833		3.578		0.938			Sxx	Syy	Sxy

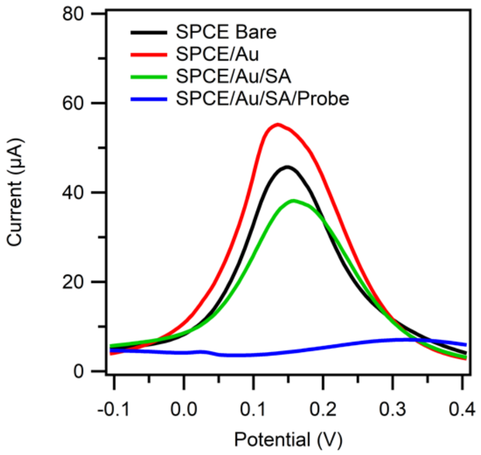

Figure S4. Differential pulse voltammogram of each before and after step of electrode modification in 10 mM $K_3[Fe(CN)_6]$ in 0.1 M KCl.

Calibration curve

Supplementary data for Fig. 4 (B) showed that the standard deviation (SD) value was 0.11%. The slopes of these calibrations were used to evaluate these parameters in terms of relative standard deviation (RSD), yielding values of 0.17% (n = 3) and 3.72% (n = 3) for reproducibility and repeatability, respectively. The recovery is 91,5652%.

Table S3. Data on the linear regression of synthetic target DNA with DPV.

Calculation of the linear regression equation

- Slope (b) = Sxy/Sxx = 4.0857
- Intercept (a) = ẏ – bẋ = 0.1736

Regression Equation: y = 4.0843x + 0.1744

- Slope and Intercept test on the confidence range of 95% (α = 0.05)

RSS = Syy–b^2Sxx

= 0.1460

$$\text{RSD} = \sqrt{\frac{\text{RSS}}{n-2}}$$

= 0.1709

- Confidence range for b on the confidence range 95% (α = 0.05; t = 2.57)

$$b \pm \frac{t(RSD)}{\sqrt{Sxx}} = 3.7220 \text{ to } 4.4494$$

- Confidence range for a on the confidence range 95% (α = 0.05; t = 2.57)

$$a \pm t(\text{RSD})\sqrt{\frac{1}{n}+\frac{x^2}{Sxx}} \quad = -0.1786 \text{ to } 0.5257$$

Because the value of a passes the zero point, the equation becomes y = bx, so the following adjustments are made:

Tabel S4. Slope value adjustment data.

No	C.DNAt (x)/ µg/mL	I (y)/µA	$(Xi)^2$	$(Yi)^2$	xy	y-predict	y-ypredict	$(y\text{-}ypredict)^2$
1	0	0	0	0	0	0.1744	-0.1744	0.0304
2	0.5	2.52	0.25	6.3504	1.26	2.2166	0.3035	0.0921
3	0.75	3.16	0.5625	9.9856	2.37	3.2376	-0.0776	0.0060
4	1	4.34	1	18.8356	4.34	4.2587	0.0813	0.0066
5	1.25	5.18	1.5625	26.8324	6.475	5.2798	-0.0998	0.0099
6	1.5	6.27	2.25	39.3129	9.405	6.3009	-0.0309	0.0009
Total	5	21.47	5.625	101.3169	23.85			0.1460
Average	0.8333	3.5783	0.9375	16.8862	3.975			

- Slope (b) $= \frac{\sum xy}{\sum xx}$ = 4.24

So, the linear equation becomes y = 4.24x

- Calculation of the limit of detection (LoD) and limit of quantification (LoQ)
- Standard deviation $(Sb) = \sqrt{\frac{\sum(y - y\ perdict)2}{n-2}}$

  = 0.191075487

- limit of detection (LoD) = 3 x sb / b = 0.13519492
- limit of quantification (LoQ) = 10 x sb / b = 0.45065

Tabel S5. Data precision, accuracy, and recovery

Target concentration (µg/mL)	Current (i) (µA)	Sb	KV (%)	Precision (%)	E (%)	Accuracy (%)	Recovery (%)
0.75	3.892<br>3.655<br>3.674<br>3.417<br>3.674<br>3.461<br>3.964	0.1178	3.3697	96.6302	8.4347	99.9367	91.5652

Precision calculation

$$KV = \frac{Sb}{\bar{\bar{x}}}\ x\ 100\%$$

= 3.3697

precision = 100% - KV

= 96.6302 %

Accuracy and Recovery calculation

$$\%\text{Error} = \frac{|\bar{x} - \mu\ |}{\mu} \times 100\%$$

= 8.4347 %

accuracy = 100% - (|ẋ-μ|*100%)

= 99.9367

recovery = (100-%E) %

= 91.5652

Mixed Real Sample Composition

Table S6. Percentage Composition of Meat (Meatballs)

	100%	50%	20%	10%	5%	1%
Chicken	5 g	1.25 g	2.75 g	3.25 g	3.5 g	3.7 g
Pork	5 g	2.5 g	1 g	0.5 g	0.5 g	0.05
Beef	5 g	1.25 g	1.25 g	1.25 g	1.25 g	1.25 g

Table S7. Peak current value from DPV measurement of mixed meat samples

	100%	50%	20%	10%	5%	1%
1	6,7490	6,8010	4,3861	3,5623	1,2500	0,0000
2	7,1101	6,8861	5,0111	3,6902	2,0011	0,0001
3	5,9800	5,8870	4,2200	4,0121	0,9902	0,0000
